# Antifouling properties of ZnO nanorods coating on micropatterned polymers

**DOI:** 10.1371/journal.pone.0357826

**Published:** 2026-09-18

**Authors:** Azhar Al-Busaidi, Sergey Dobretsov, Htet Htet Kyaw, Myo Tay Zar Myint

**Affiliations:** 1 Department of Marine Science and Fisheries, College of Agriculture and Marine Sciences, Sultan Qaboos University, Muscat, Oman; 2 UNESCO Chair in Marine Biotechnology, Centre of Excellence in Marine Biotechnology, Sultan Qaboos University, Muscat, Oman; 3 Nanotechnology Research Center, Sultan Qaboos University, Muscat, Oman; 4 Department of Physics, College of Science, Sultan Qaboos University, Muscat, Oman; KIST: Korea Institute of Science and Technology, GERMANY

## Abstract

Biofouling remains a major challenge for submerged materials in marine and industrial environments, reducing performance and increasing maintenance costs. In this study, zinc oxide nanorod (ZnO NR) coatings with dual-scale surface roughness were fabricated on three distinct 3D micropatterned surfaces, designated as D1, D2, and D3. The antifouling performance of the engineered surfaces was evaluated against the bacterium *Escherichia coli* and the diatom *Amphora* sp. under laboratory flow conditions, while acute toxicity was assessed using larvae of *Litopenaeus vannamei*. Fourier transform infrared (FTIR) spectroscopy confirmed the presence of Zn–O stretching vibrations, particularly on the ZnO NR-coated D2 and D3 surfaces. Water contact angle measurements showed that the ZnO NR-coated surfaces were superhydrophobic (≈150–165°) because of their hierarchical micro/nanostructures, whereas the non-coated surfaces exhibited moderate wettability (≈80–90°). ZnO coating reduced bacterial attachment by 60.3%, 48.8%, and 5.8% on the D1, D2, and D3 surfaces, respectively. Diatom coverage was reduced by 9.9%, 72.9%, and 71.8% on the corresponding ZnO-coated surfaces. The enhanced antifouling performance was associated with the combined effects of Zn^2+^ ions, ROS generation, surface wettability, and micro/nanotopography. The non-coated D1 and D2 surfaces, together with the ZnO-coated D1 surface, exhibited no significant toxicity toward shrimp larvae. In contrast, the ZnO-coated D3 surface exhibited relatively low antifouling performance and higher larval toxicity. Among the tested surfaces, the ZnO-coated D1 micropattern demonstrated the best overall performance by combining high antifouling efficacy with low toxicity. Post-characterization SEM analysis confirmed that the ZnO nanorod coatings remained structurally intact after biological testing, indicating good coating stability under the experimental conditions. These findings demonstrate that combining ZnO nanorod coatings with micropatterned surface topography is a promising strategy for antifouling applications. However, further long-term field evaluations and comprehensive ecotoxicological studies are required to assess the environmental performance of these coatings.

## 1. Introduction

Marine biofouling is the accumulation and growth of undesired biological organisms on submerged surfaces [1]. It is a multistage process that begins with surface conditioning, followed by the development of microbial biofilms composed of bacteria, diatoms, and microalgae, and, finally, by the settlement of sessile macrofouling organisms, such as barnacles, hydroids, mussels, sponges, and bryozoans [2].

Biofouling has serious issues for both industry and the navy in terms of reduction of boat and ship speed, clogging water intakes and heat exchangers, changing buoyancy, and overgrowing aquaculture nets [3]. Additionally, biofouling organisms enhance corrosion, shear stress, and drag, resulting in higher fuel consumption and increased CO_2_ output by ships. Worldwide, nations spend more than $5 billion a year trying to prevent and deal with biofouling [4,5]. Overall, there is a significant need for the development of materials that resist and prevent fouling. These materials are called antifouling materials [6].

Currently, two main strategies are used to combat biofouling: biocide-based antifouling coatings and non-toxic, foul-release coatings [3]. Biocidal coatings inhibit organism growth through the release of toxic compounds. Foul-release coatings are hydrophobic and possess low surface energy, minimizing organism adhesion and allowing easy detachment under hydrodynamic forces [7]. However, concerns regarding toxicity and environmental impact have driven the development of non-toxic antifouling strategies that modify the physical or chemical properties of surfaces or use environmentally benign materials [8–10].

Antifouling properties of marine organisms inspired engineers to develop biomimetic antifouling solutions, including superhydrophobic and microtopography coatings [11]. Sharks and mollusks prevent the settlement of biofouling organisms due to their micro-structured surfaces [12–14]. Biomimetic replication of these textures has been shown to influence the settlement of algal spores and barnacles, suggesting that surface patterning can physically deter biofouling [15,16]. However, the main limitations of such bioinspired antifouling coatings are their short service life and low specificity against different fouling organisms [11]. Therefore, the development of new antifouling coatings should integrate both biomimetic and traditional approaches.

Recently, nanotechnology has been used to provide a solution for water treatment and prevention of biofouling [17,18]. Nanotechnology uses materials having nanoscale dimensions ranging from a few nanometers to around 100 nanometers in any or all dimensions [19]. Pure nanoparticles are challenging to produce unless the entire process is carried out in an inert environment. Laser ablation, thermolysis, chemical reduction, and polyol synthesis are some of the many processes used to create nanoparticles. Chemical reduction is frequently chosen among these methods because it is easy, cheap, and efficient [11]. Metal oxide nanoparticles have drawn considerable interest due to their broad-spectrum antimicrobial (i.e., antibacterial, antiviral, and antifungal) activity. However, nanoparticles compared to other nanostructures, such as nanorods (NRs), have higher toxicity to different marine organisms [19]. Additionally, NRs can be attached to the surfaces, thus limiting their release into the environment [20]. Zinc oxide nanorods (ZnO NRs) exhibit strong antimicrobial and antifouling activity due to their photocatalytic generation of reactive oxygen species (ROS) under light exposure [21]. ROS damage microbial membranes and prevent initial colonization of biofouling organisms [22,23]. When integrated into polymeric or resin-based coatings, ZnO NRs enhance surface roughness, reduce adhesion strength, and combine micro/nano-scale topographic effects with photocatalytic self-cleaning, offering a promising alternative to conventional biocidal coatings with relatively low acute toxicity under the tested conditions [6,18].

Recent advances in antifouling materials have increasingly focused on hybrid systems that combine polymeric substrates with nanoscale functional materials to control biological adhesion through both physical and chemical mechanisms. For example, nano-protrusive gold nanoparticle-hybridized polymer thin films have been developed as sensitive and antifouling biosensor platforms, where nanoparticle-induced surface roughness improves sensing performance while reducing nonspecific bio-adhesion [24]. Similarly, horizontally ordered nanofibrous hydrogel films have demonstrated that nanoscale surface architectures can effectively regulate cell–surface interactions and biological responses [25]. In membrane applications, nanoporous antifouling polymer coatings prepared from amphiphilic block copolymer blends have been shown to mitigate fouling through tailored surface chemistry and morphology [26]. Other nanoparticle-based systems, such as Ag/Fe co-doped hydroxyapatite materials and bioinspired Fe-based amorphous coatings, rely primarily on antimicrobial activity or dual killing–resisting mechanisms to suppress biological attachment [27,28]. In contrast, the present study integrates ZnO nanorods with micropatterned polymer surfaces to generate hierarchical micro/nano-scale structures that combine topographical antifouling effects with photocatalytic ROS generation, Zn² ⁺ ion release, and wettability modification. This multifunctional design provides a distinct strategy for controlling bacterial and algal settlement while simultaneously evaluating biological compatibility through toxicity assessment.

The main aims of this study were to combine nanotechnology-based coatings with biomimetic solutions and to develop dual roughness ZnO nanorods (ZnO NRs) antifouling coatings. ZnO NRs were developed on three different micropatterned surfaces, and their antifouling performance was investigated in laboratory experiments. The specific objectives of this study were: (1) design and fabricate ZnO NRs-coated 3D printed patterned surfaces; (2) to investigate the antifouling properties of ZnO NRs coatings under dynamic conditions using a Gram-negative bacterium (*Escherichia coli*) and a microalga (*Amphora* sp.); (3) to study the toxicity of created coatings using shrimp larvae of *Litopenaeus vannamei;* and (4) to investigate leaching of Zn^2+^ ions and formation of ROS from the coatings. *E. coli* was selected as a representative freshwater-associated bacterium and model microorganism to evaluate the antibacterial activity of the fabricated surfaces under freshwater conditions. In parallel, the marine diatom *Amphora* sp. and shrimp larvae were used to assess the antifouling performance and biological compatibility of the same coatings under seawater conditions. This experimental design enabled the evaluation of the coatings across both freshwater and marine environments, reflecting the broad applicability of antifouling technologies.

## 2. Materials and methods

### 2.1 Chemicals

Zinc acetate dehydrate (Zn (CH_3_CO_2_)_2_.H_2_O), zinc nitrate hexahydrate (Zn(NO_3_)_2_ 6H_2_O), and hexamethylenetetramine (C_6_H_12_N_4_) were obtained from Sigma-Aldrich, USA. Sodium hydroxide (NaOH) was procured from Honeywell, Germany. Absolute ethanol (C_2_H_5_OH) and terephthalic acid were obtained from Merck, Darmstadt, Germany. Standard deionized water (DI) was used throughout the experiment. Nutrient broth was obtained from Sigma-Aldrich.

### 2.2 Preparation of micropatterned surfaces

**Fig 1:**
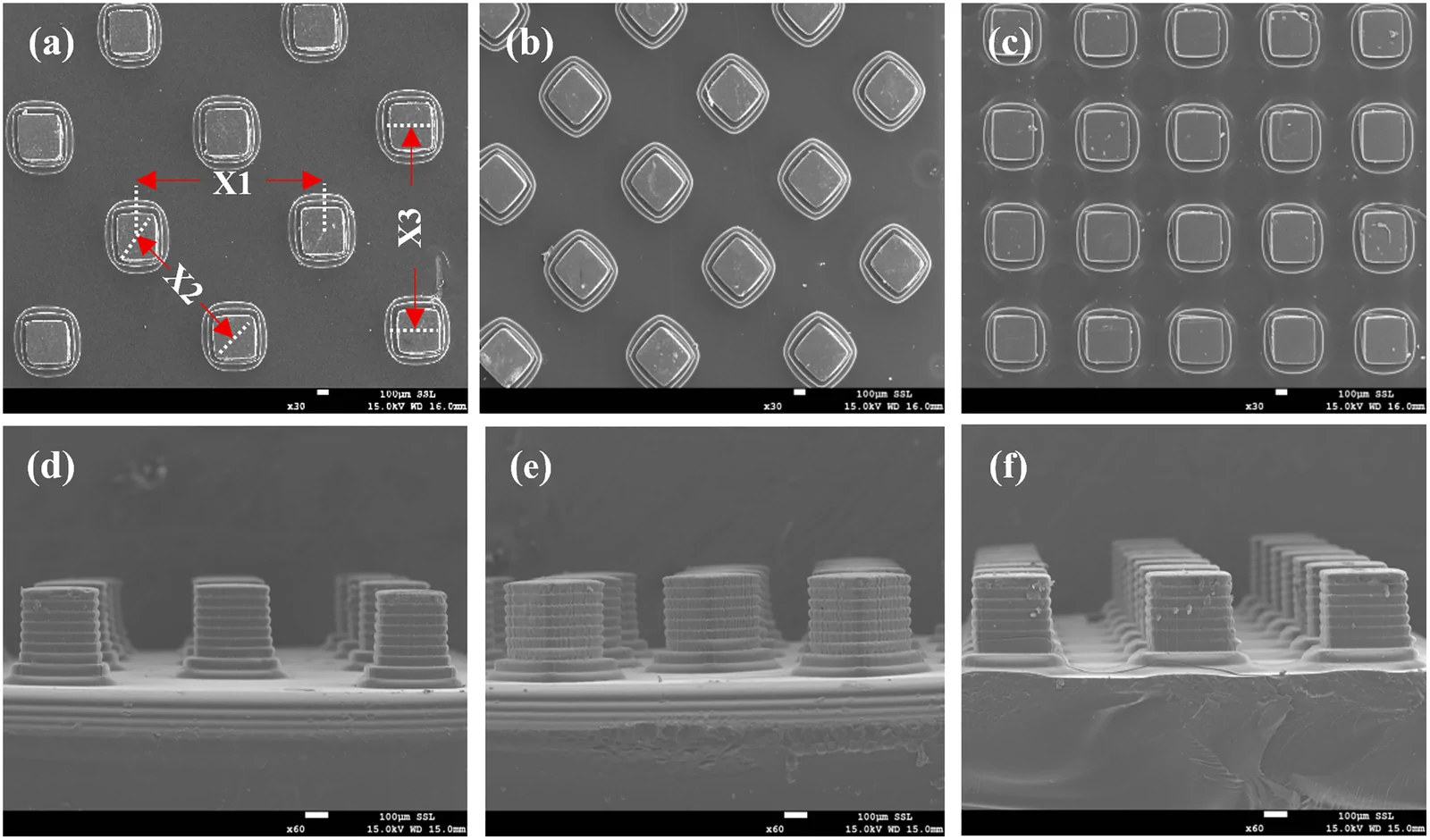
The scanning electron microscope (SEM) images for different polymer micropattern (without ZnO coating) and their cross sections: (a,d) D1, (b,e) D2, and (c,f) D3.

First, three distinct micropattern geometries, denoted as pattern 1 (D1), pattern 2 (D2), and pattern 3 (D3), were designed using standard computer-aided design (CAD) software with basic mechanical 3D modeling tools. The micropattern shapes were selected based on the previous biomimetic surfaces. The three micropatterns were designed with identical feature height, width, and shape but different feature spacing to evaluate the influence of spacing on antifouling performance while minimizing the effects of other geometric parameters. The digital models were prepared for vat photopolymerization printing [24]. The micropatterned panels were fabricated using an Anycubic Craftsman resin-based LCD 3D printer (Anycubic Photon M3 Premium 8K, Shenzhen Anycubic Technology, China) via vat photopolymerization technology. To start the printing, support structures were automatically generated where required during slicing. Anycubic craftsman resin is composed of 30–60% polyurethane acrylate, 10–40% acrylate monomer, and 2–5% photoinitiator. The mixture served as the photopolymer material. The liquid resin was selectively cured layer by layer under ultraviolet (UV) light, initiating free-radical polymerization. These layers crosslinked the acrylates, transforming the liquid into a solid polymer resin. Post-printing, excess uncured resin was initially removed at the printing facility using Anycubic Wash and Cure equipment, which combines alcohol-based washing and air drying. The area of the 3D printing substrate for each pattern is 7.2 cm × 2.4 cm, and the detailed shapes and dimensions are shown in Fig 1 and Table 1. The patterns were cleaned by sonication in a soap solution for 15 min. Then, the panels were washed with tap water and cleaned by sonication in deionized water (DI) for 15 min, followed by drying in an oven at 70 °C for 24 h.

**Table 1:** Dimensions and total surface area in μm^2^ of 3D printed micropatterns.

Parameters	Pattern 1 (D1)	Pattern 2 (D2)	Pattern 3 (D3)
Height (μm)	390.9 ± 7.2	390.6 ± 2.9	390.2 ± 1.9
Width (μm)	388.6 ± 3.0	350.2 ± 4.4	369.6 ± 5.1
Length (μm)	389.8 ± 1.0	375.7 ± 5.3	372.5 ± 4.4
Horizontal distance (μm) (X1)	1588.6 ± 3.4	1386.2 ± 2.5	849.0 ± 5.0
Diagonal distance (μm) (X2)	1127.1 ± 1.8	970.2 ± 1.7	1200.6 ± 2.2
Vertical distance (μm) (X3)	1705.5 ± 3.9	1386.3 ± 2.1	862.3 ± 2.9
Total effective surface μm^2^	2.548 × 10^9^	2.733 × 10^9^	3.805 × 10^9^

### 2.3 Synthesis of ZnO nanoparticles, seeding and ZnO nanorods growth process

The growth process of ZnO nanorods (NRs) was followed as described by Al-Belushi et al. [29], with slight modifications. First, ZnO nanoparticles were prepared as follows. 4 mM of Zn (CH_3_CO_2_)_2_.H_2_O and 4 mM of NaOH were dissolved separately in 40 ml of C_2_H_5_OH under continuous stirring for 30 minutes. Then, the prepared 4 mM NaOH solution was mixed with 40 ml of C_2_H_5_OH and continued stirring for another 30 minutes. Two solutions of Zn (CH_3_CO_2_)_2_.H_2_O and NaOH were mixed and then kept in a preheated water bath at 60 °C for 2 h to form ZnO nanoparticles. The reaction was stopped after 2 h, and the ZnO nanoparticle solution was cooled to room temperature for further use. For each micropattern surface, 10 ml of ZnO nanoparticle solution was sprayed while heating at 65 °C. For the growth of ZnO NRs, an equimolar solution of 10 mM Zn(NO_3_)_2_ 6H_2_O and C_6_H_12_N_4_ in DI water was used. The pre-seeded micropattern surfaces were properly arranged with upside down position in glass Petri dishes and 100 ml from each prepared solution was added, then placed in a microwave for 45 min at 180 W. Every 45 min, the solution was replaced with a new precursor solution at the same ratio. The growth was carried out for 135 min. At the end, the samples were washed with DI water and placed in an oven at 90 °C until further use. These ZnO NRs-coated micropattern surfaces are named as D1 ZnO, D2 ZnO, and D3 ZnO.

### 2.4 Characterizations of micropattern surfaces

#### 2.4.1 Scanning Electron Microscopy (SEM).

Surface morphology and elemental composition of coatings (before and after the antifouling experiment) were observed by field emission scanning electron microscopy (FESEM, JEOL JSM-7800F, Japan) equipped with energy-dispersive X-ray spectroscopy (EDXS) (Oxford Instruments, UK). SEM images were obtained for the control surfaces (without ZnO NRs) and the patterns with ZnO NRs.

The total effective surface area of the micropatterned panels is calculated according to the following equation:


$$\begin{array}{c} \text{Total effective surface area }({\text{mm}}^{\text{2}})={\text{A}}_{\text{panel}}+\text{4}(\text{h}\times \text{a})\times \text{N} \end{array}$$

where A_panel_ is the 3-D panel area (mm²), h is the height of the micropatterns (mm), a is the side length of each micropattern (mm), and N is the number of micropatterns within the panel region. The term 4(h × a) accounts for the sidewall surface area contributed by each square micropattern.

#### 2.4.2 Attenuated Total Reflection Fourier Transform Infrared spectroscopy (ATR-FTIR).

Attenuated Total Reflection Fourier Transform Infrared (ATR-FTIR) spectroscopy was used to analyze the surface functional groups of the control and ZnO NRs-coated micropattern surfaces. The spectra were collected using a PerkinElmer Spectra One spectrometer (USA) in the wavenumber range of 4000–550 cm^−1^ with a spectral resolution of 4.0 cm^−1^.

#### 2.4.3 Surface Wettability.

To determine the surface wetting nature of ZnO NRs coated and uncoated 3D surfaces, the water contact angle (WCA) was measured using a Theta Lite attention tensiometer (Biolin Scientific, Sweden). Briefly, a drop of 5 μl DI water was placed on the surface, and a picture of the water droplet was taken by the camera. The contact angle was measured using ImageJ software (http://rsbweb.nih.gov/ij/, NIH, USA). Three repeated measurements were carried out and the average WCA for each surface was reported.

#### 2.4.4 Reactive oxygen species test.

To evaluate ROS generation from the ZnO NR-coated surfaces (D1 ZnO, D2 ZnO, and D3 ZnO), a 0.5 mM terephthalic acid (TA) solution was prepared in 0.2 mM aqueous NaOH. Panel samples (2 cm²) were immersed in 5 mL of the TA solution and exposed to solar-simulated irradiation at 1000 W/m² (Sciencetech, Canada). After 30 min of visible-light exposure, the TA fluorescence was recorded using a photoluminescence spectrometer (PerkinElmer LS 55, United States) at an excitation wavelength of 315 nm.

### 2.5 Antifouling test

#### 2.5.1 Antibacterial test.

The antibacterial activity of ZnO NRs-coated and uncoated micropattern surfaces was tested in the laboratory. All experiments were conducted in triplicate. First, the Gram-negative bacterium *Escherichia coli* (ATCC 25922) was incubated at 37 °C for 24 h in a sterile nutrient broth (Sigma- Aldrich, St. Louis, MO, USA). Plates with six different patterns coated with ZnO NRs (D1 ZnO, D2 ZnO, and D3 ZnO) and not coated (D1, D2, and D3) were used in this experiment. Second, using double adhesive tape, the panels were randomly attached vertically to the walls of the 1L glass beaker. Third, 0.5 L of sterile fresh water was mixed with 1 mL of bacterial culture (optical density = 0.53) and transferred into a sterile beaker. The culture solution was kept under continuous stirring (800 rpm) at 26 °C for 24 h under light at an irradiance of 3.54 klx. Finally, at the end of the experiment, the samples were removed from the beaker and stored at −80 °C for subsequent quantification of attached bacteria.

Bacterial abundance on the surface of samples was determined using an epifluorescence microscope following the protocol by Muthukrishnan et al. [30]. Samples were stained with DAPI and observed under an epifluorescence microscope (Olympus BX51, USA) at 1000 × magnification, using DAPI’s excitation/emission wavelengths (λE_x_ = 359 nm, λE_m_ = 441 nm). Bacterial cells were counted in 10 randomly selected fields of view, and the average count was used to estimate the total cell density on each panel.

#### 2.5.2 Antialgal test.

Previously*,* the diatom *Amphora* sp. was isolated from fouled substrata in the Sea of Oman. Before the experiment, these diatoms were cultured for 10 days in F2/2 medium under a 12/12 h light/dark cycle at a light intensity of 1.5–2 klx. For the experiment, six treatments (three coated with ZnO NRs and three uncoated) were placed in a beaker. Uncoated panels were used as a control. The plates were attached using double-sided adhesive tape to the walls of the beaker. A 0.1 L inoculum of *Amphora* sp. culture (0.051 OD) was added to a 1 L beaker containing sterile seawater, which was then transferred to the treatment beakers and maintained under continuous stirring (800 rpm) at 26 °C for 24 h. Illumination was provided by a luminescent lamp at a light intensity of 3.5 klx. All experiments were conducted in triplicate. At the end of the experiment, the plates were Finally, at the end of the experiment, the samples were removed from the beaker and stored at 4 °C for counting of attached diatoms.

Images of diatoms attached to the textured patterns were captured from three randomly selected areas using an Axiocam HRc camera (Carl Zeiss AG, Jena, Germany). The percentage of diatom coverage was quantified using ImageJ software (http://rsbweb.nih.gov/ij/, NIH, USA). Briefly, the fouled regions in each image were first cropped and calibrated. Then, color thresholds were manually adjusted to enhance contrast between fouled and unfouled areas. The fouling area was subsequently measured, and the percentage of cover was calculated as the ratio of fouled area to the total area for each image.

### 2.6 Toxicity testing

The toxicity of ZnO NRs-coated and non-coated micropattern surfaces was investigated in the laboratory. Shrimp larvae of *Litopenaeus vannamei* (Boone, 1931) with a size of 1 cm were used in this experiment. This shrimp was selected because it is a main marine aquaculture species in Oman. Five larvae with 3 ml of sterile seawater were placed in a single Petri dish (diameter = 90 mm), which contained one plate with ZnO NRs coated and uncoated surfaces. A control treatment consisting of shrimp larvae maintained in sterile seawater without exposure to any micropatterned or ZnO-coated surface was included to determine baseline survival under the experimental conditions. The experiment was carried out for 24 h at 25 °C. After 24 h, the number of alive larvae was counted using a microscope. The mortality was calculated as a percentage to evaluate toxicity. All experiments were conducted in triplicate.

### 2.7 Leaching test

To conduct a leaching test, 2 x 2 cm of ZnO NRs-coated and uncoated substrates were immersed in 100 ml of deionized water. Ten ml from each sample were collected after 24 h. Inductively coupled plasma-optical emission spectrometry (ICP-OES, Perkin Elmer 8000 DV, Waltham, MA, USA) was utilized to analyze the concentration of Zn^2+^ ions in the water to estimate the leaching of ions from the coating.

### 2.8 Data analysis

All biological experiments were performed using three independent replicate samples (n = 3) for each treatment. Means and standard errors for bacterial count, diatom percentage of cover, and shrimp percentage of mortality were analyzed using analysis of variance (ANOVA), followed by Tukey’s post-hoc test or pairwise comparisons. Factors included coating (with ZnO NRs and without them) and treatment (different micropatterns D1-D3). Prior to the analysis, the assumptions of normality and homogeneity of variances were verified using the Shapiro–Wilk test. In all cases, the significant difference was *P* < 0.05. All descriptive statistics and statistical analyses were conducted using R studio version 4.3.1 (2024), and graphical representations were created using OriginPro 2023 software.

## 3. Results and discussion

Previous studies suggested that the micropatterned morphology plays a significant role in enhancing antifouling properties [31]. However, the main limitation of such micropatterned antifouling coatings is low specificity against different fouling organisms. In order to overcome this problem, in this study, different micropatterned surfaces (D1, D2 and D3) were covered with ZnO nanorods (NRs). The antifouling performance of such coatings was evaluated using the bacterium *E. coli* and the diatom *Amphora* sp. in the flow laboratory experiments. The toxicity of the coatings was studied using shrimp larvae as a test organism.

### 3.1 Characterizations of micropattern surfaces

The dimension of the 3D printed micropattern surface D1 is found to be 388.6 ± 3.0 μm x 389.8 ± 1.0μm and the height of 390.9 ± 7.2μm (Fig 1a and 1d), D2 is 350.2 ± 4.4μm x 375.7 ± 5.33μm with the height of 90.6 ± 2.9μm (Fig 1b and 1e), and D3 is 369.6 ± 5.1μm x 372.5 ± 4.4μm with the height of 390.2 ± 1.9μm (Fig 1c and 1f). The horizontal distance between each micropillar is estimated at 1588.6 ± 3.4μm for D1, 1386.2 ± 2.5μm for D2, and 849.0 ± 5.0μm for D3, and the diagonal distance between each micropillar is estimated at 1127.1 ± 1.8μm for D1, 970.2 ± 1.7μm for D2, and 1200.6 ± 2.2μm for D3. The results indicated that total surface area for D1 is 2.548 × 10^9^ μm^2^, D2 is 2.733 × 10^9^ μm^2^and D3 is 3.805 × 10^9^ μm^2^ (Table 1).

**Fig 2:**
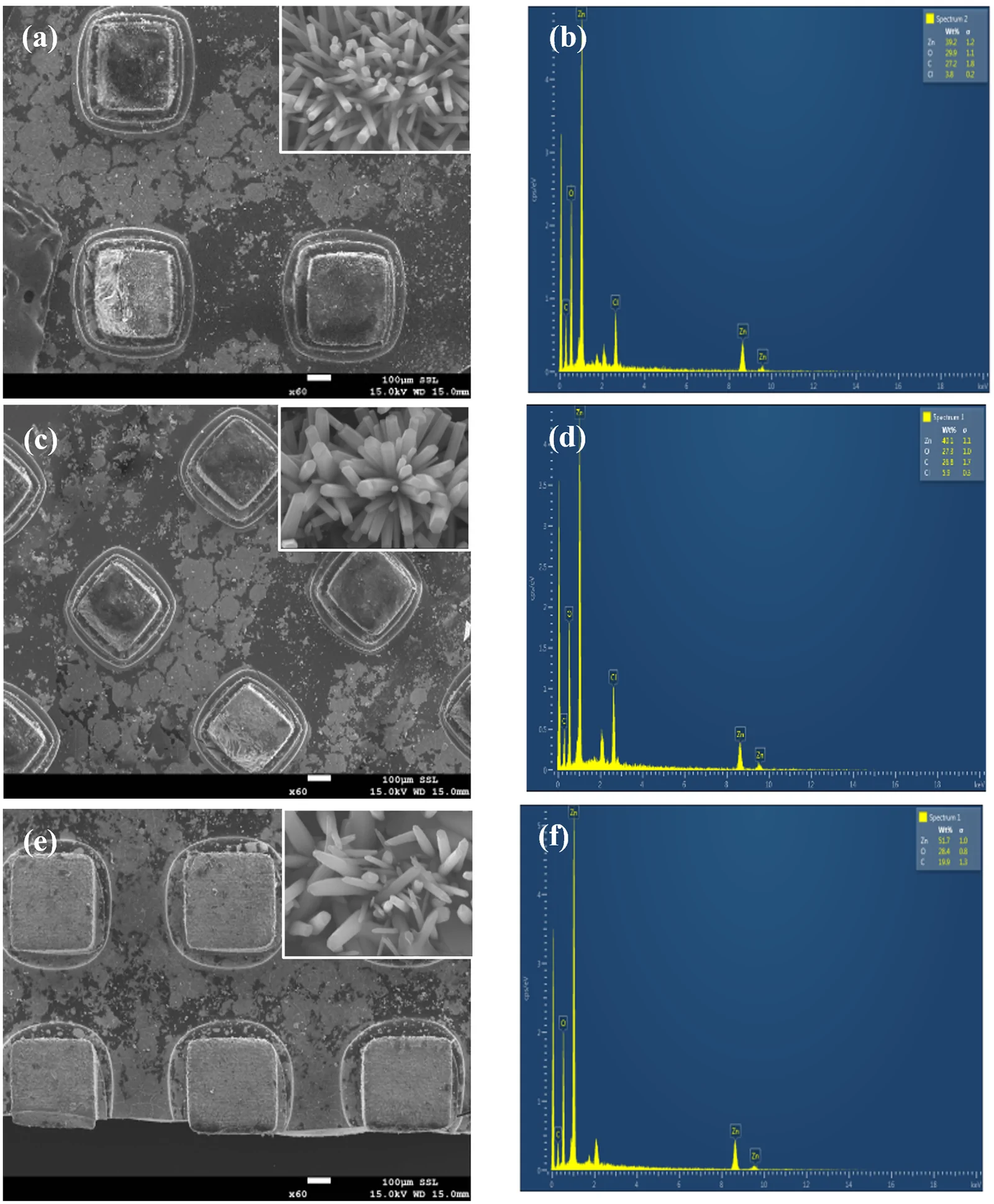
The scanning electron microscope (SEM) images for ZnO NRs-coated micropattern surfaces and energy-dispersive X-ray spectroscopy in different treatments: (a, b) D1 ZnO, (c, d) D2 ZnO, and (e,f) D3 ZnO. The insets show dense growth of ZnO NRs on surfaces.

ZnO NRs were successfully deposited onto the 3D-printed micropattern surfaces, as shown in Fig 2. The elements carbon (C) and oxygen (O) were detected on the surface of the micropatterns which are made of polymers, originating from the base materials. The ZnO NRs appeared in a densely grown fashion on the patterned surfaces (Fig 2). Although the concentration of the ZnO nanorod (NR) growth solution was identical for all micropatterned surfaces, the density of the ZnO NRs varied among the D1, D2, and D3 patterns. This variation is likely attributable to the non-uniform deposition of ZnO nanoparticles during the seeding process. EDXS analysis showed that the Zn content ranged from 27–48% for D1, 30–33% for D2, and 25–29% for D3. Please note that the EDXS measurements were performed at three randomly selected locations on each sample, and the Zn content range is reported.

The insets in Fig 2a, 2c and 2e show the characteristic hexagonal morphology of the ZnO nanorods grown on the micropatterned surfaces, with diameters ranging from approximately 50–190 nm. The ZnO nanorods exhibited a morphology consistent with that reported in our previous studies using the same hydrothermal growth method [19–21]. Although quantitative measurements of nanorod dimensions and surface coverage were not performed in the present study, the SEM observations confirmed the successful growth of ZnO nanorods on all micropatterned surfaces, with differences in nanorod density among the three geometries.

### 3.2 FTIR spectroscopy

**Fig 3:**
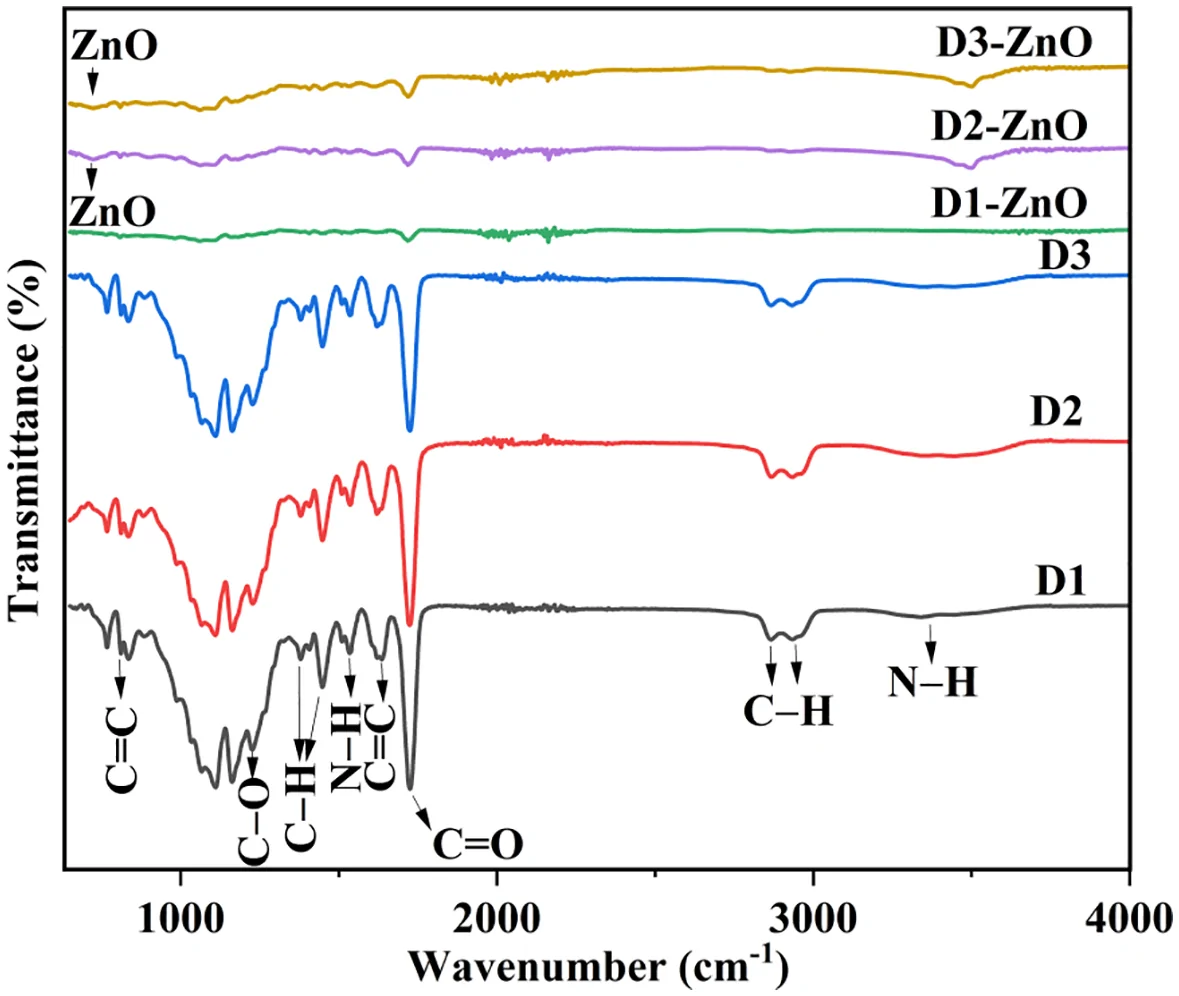
FTIR spectra of uncoated (D1, D2, and D3) and coated (D1 ZnO, D2 ZnO, and D3 ZnO) sample surfaces.

The FTIR spectra of micropattern surfaces uncoated (D1, D2, and D3) and coated with ZnO (D1 ZnO, D2 ZnO, and D3 ZnO) are shown in Fig 3. Uncoated sample surfaces exhibit several absorption peaks in the region of ~500–1800 cm^−1^, along with weak bands above 2000 cm^−1^. Prominent peaks in the range of 1000–1600 cm^−1^ can be attributed to functional groups such as C–O stretching, C = C stretching, and C–H bending vibrations from the polymer material [32–38].

In comparison, samples coated with ZnO surfaces (D1 ZnO, D2 ZnO, and D3 ZnO) display spectra with reduced peak intensity (Fig 3). The characteristic absorption bands appear broader and flatter, which indicates the influence of the ZnO NRs coating. The smoother spectral profiles of the coated samples suggest that ZnO deposition partially masks or overlaps the organic signals originating from the underlying material. In the low-wavenumber region around 600–800 cm^−1^, the presence of Zn–O stretching vibrations is observed.

Analysis of the chemical and physical properties of antifouling coatings is important in order to identify how different surface compositions and structures will influence antifouling mechanisms related to wettability, ion release and photo-catalysis and ultimately affect the ability of microorganisms to attach to surfaces and form biofilms [39]. Therefore, FTIR Spectroscopy is an essential tool for confirming that antifouling coatings are formed and identifying functional groups present on the coatings that have contributed to their antifouling-related surface properties.

A distinct absorption band appeared in the region of 600–800 cm^−1^, which corresponds to the Zn–O stretching vibration, confirming the successful formation of ZnO nanorods on the surface of the micropatterns. The presence of this Zn–O band agrees with previously reported values for ZnO nanostructures and ZnO/polyurethane composites [40–42]. The intensity of the Zn–O peak was obvious in the D2 ZnO and D3 ZnO samples compared to D1 ZnO. The largest surface area of the three micropatterns was in D3, with D2 being second and D1 least. All three micropatterns provide more nucleation sites for ZnO nanorods to grow. The relationship between surface area available for ZnO to nucleate and form crystals and how that relates to the amount of nanostructure grown is supported by literature that indicates both the nucleation and development of crystals of ZnO are significantly influenced by the characteristics of the surface of the substrate’s surface upon which it forms [17,43]. In contrast, D1 ZnO exhibits a weak intensity of the Zn–O peak, likely due to the inhomogeneous distribution of ZnO NRs, where some areas have a lower density of ZnO NRs on the 3D surface, as observed in the SEM image in Fig 2a. This could be due to the non-uniform deposition of ZnO nanoparticles during the seeding process. From the EDXS analysis, the broader Zn content distribution in D1 further confirmed the inhomogeneous distribution of ZnO NRs, resulting in a lower Zn-O peak intensity.

D1, D2, and D3 exhibited characteristic polymer absorption peaks. The bands around 2920–2850 cm^-1^ correspond to C–H stretching vibrations of aliphatic chains, while the strong band near 1725 cm^-1^ is assigned to C = O stretching of nonhydrogen-bonded carbonyl groups of both polyurethane and acrylate compounds [44]. The band at 1634 cm^-1^ and at 810 cm^-1^ correspond to the C = C stretching vibrations of the methacrylate group [45]. The band at 1535 cm^-1^ is attributed to the N-H bending vibration of the amide II band, while the weak broad band between 3200−3700 cm^-1^ is assigned to the N-H stretching vibration of the polyurethane backbone. The bands at 1450 cm^-1^ and at 1374 cm^-1^ are assigned to the C-H bending vibrations of CH_2_ and CH_3_ groups [46,47]. Additional peaks observed between 1350 and 1000 cm^-1^ are attributed to vibrational modes of C–O–C ester bonds, where a weak urethane C-O stretching band is included at 1227 cm^-1^ [44]. The appearance of Zn–O stretching bands exclusively in the coated samples demonstrates that ZnO nanorods were successfully grown and integrated on micropattern surfaces without altering the main functional groups of polyurethane.

### 3.3 Wettability properties

**Fig 4:**
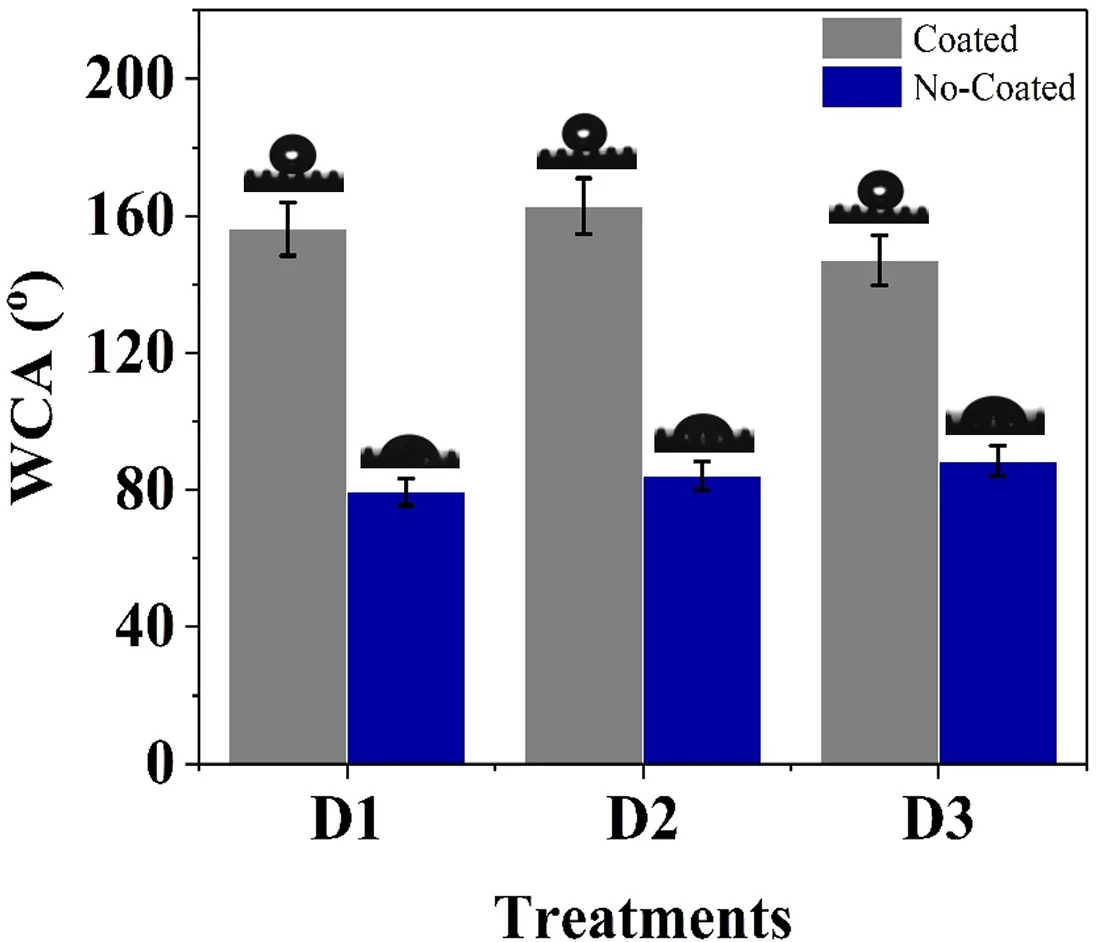
Water contact angles (WCA) of ZnO NRs coated and non-coated D1, D2 and D3 micropatterned samples.

The water contact angle results on uncoated (D1, D2, and D3) and coated (D1 ZnO, D2 ZnO, D3 ZnO) surfaces are shown in Fig 4. Generally, coated surfaces had higher water contact angles (WCA) compared to uncoated ones. The highest WCA of 163° was observed for D2 ZnO followed by D1 ZnO with a WCA of 156°, while the lowest was found for D3 ZnO with a WCA of 147°. However, for non-coated samples, the highest water contact angle was reported for D3 with a WCA of 89° followed by D2 (WCA = 84°), while the lowest one was found for D1 with approximate WCA of 79° (Fig 4).

Our study showed that ZnO-coated surfaces (D1 ZnO, D2 ZnO, and D3 ZnO) exhibited superhydrophobic nature, with WCA values that ranged from 150° to 165°. On the contrary, uncoated surfaces exhibited a partially hydrophobic nature with WCA values ranging from 80° to 90°. This transition from hydrophobic to superhydrophobic surface nature was due to both the surface roughness of the microstructure of micropatterns and the presence of ZnO NRs. This demonstrates the synergistic effects of micro/nano-scale hierarchical surface roughness from ZnO nano-scale structures [48].

When ZnO NRs are formed on top of the patterned substrate, the nanorods increase the surface roughness as well as the development of air-trapping pockets between the water drops and the solid surface. Cassie-Baxter wetting theory explained the reduction of solid-liquid contact, which led to an increase in WCA [43]. Previously, we have demonstrated that ink-jetted micropattern ZnO microrods turned the glass surface into a superhydrophobic surface [40]. In this case, both ZnO NRs and micropattern features work synergistically to make a water-repellent effect, which is typical of many hierarchical structure superhydrophobic coatings [17].

Among the coated samples, D2 ZnO exhibited the highest WCA, indicating that the micropattern of D2 showed the ideal trade-off to achieve effective air pocket retention through an appropriate combination of structural spacing, height and the presence of nanorods to create the superhydrophobic nature. Similarly, Zhang et al. [49] have reported that the geometric structure of the surface has a significant influence on modifying wetting behavior as long as the nanostructure is present. The improvement in hydrophobicity demonstrated by the increased WCA confirmed that the ZnO NRs were successfully grown and further highlighted how surface wetting characteristics could be manipulated by the addition of micro- and nano-structures. This micro/nano structural formation plays an important role in preventing biofouling in addition to the photocatalysis process, which will be discussed in a later section.

### 3.4 Antifouling assay

#### 3.4.1 Antibacterial test.

**Fig 5:**
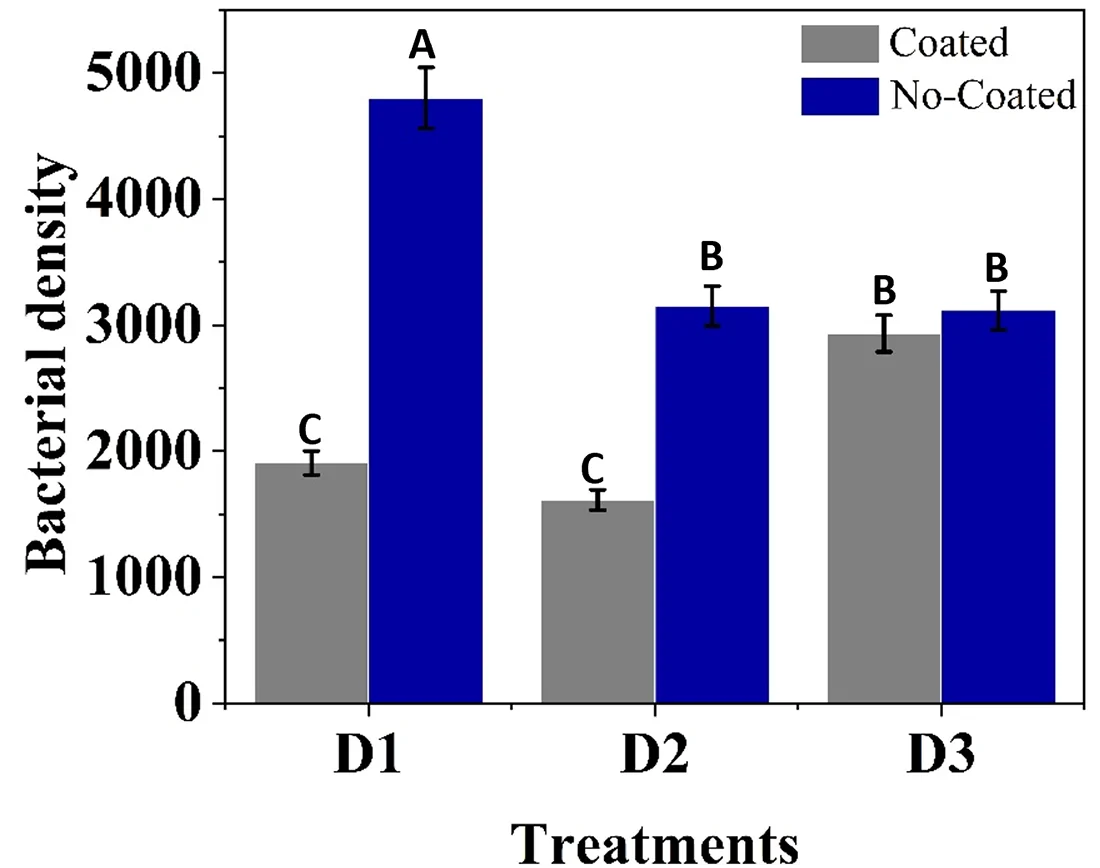
The mean ± SD of the bacterial density (cells mm^-2^) of ZnO NRs coated and non-coated D1, D2 and D3 micropatterned samples. Different letters above the bars indicate significant differences by the Tukey HSD test (*P* < 0.05).

The density of *E. coli* bacteria on the plates varied among treatments after 24 h (Fig 5). Coating significantly affected bacterial density (ANOVA, *P* < 0.0001) (Table S1 in S2 File). Usually, coated samples had higher antibacterial activity compared to non-coated ones (Fig 5). The highest bacterial densities were observed on non-coated D1 plates, whereas the lowest densities were detected in coated D1 ZnO and D2 ZnO samples. In contrast, bacterial densities were nearly identical for uncoated D3 and coated D3 ZnO samples. ANOVA analysis showed that ZnO coating is the primary factor responsible for the reduction in bacterial density, as demonstrated by the highly significant coating effect (*p* < 0.0001, Table S1 in S2 File). In contrast, the effect of micropattern geometry (treatment) was not statistically significant (*p* > 0.05). The interaction between treatment and coating type was significant (ANOVA, *P* = 0.0062) (Table S1 in S2 File). This indicates that micropattern geometry modulates the antibacterial performance of the ZnO coating rather than acting as an independent antibacterial factor. Tukey post hoc tests revealed significant (*p* < 0.05) differences between bacterial densities of coated and non-coated samples (Fig 5). Bacterial densities in the D1 ZnO, D2 ZnO, and D3 ZnO samples were lower by 60.3%, 48.8%, and 5.8%, respectively, compared to the corresponding non-coated treatments D1-D3 (Fig 5).

Our study demonstrated that there were differences in the antibacterial performance of different coated and non-coated microtopography surfaces. The ZnO-coated D1 (D1 ZnO) and D2 (D2 ZnO) samples exhibited the lowest levels of bacterial fouling, whereas the antifouling performance of the ZnO-coated D3 (D3 ZnO) surface was less pronounced. These findings indicate that the ZnO NR coating is the primary factor contributing to the antibacterial activity of the micropatterned surfaces, while the effectiveness of the coating is influenced by the underlying surface geometry. Overall, the present results suggest that antifouling efficacy is determined by the combined effects of microtopography, surface wettability, reactive oxygen species (ROS) generation, and Zn² ⁺ release.

Surface wettability has significant implications for the antifouling behavior of surfaces. Based on the current results, surface wettability does not appear to account for all observed biological responses, especially concerning bacterial adhesion. Similar results have been observed earlier [10,50]. Although D3 ZnO had a 1.8-fold higher water contact angle (WCA) than the D3 sample, the amount of bacterial adhesion on both samples was similar. This indicates that, for bacteria, the combination of micropattern geometry, feature spacing, and the presence of ZnO NR coating has a stronger effect and may overcome surface wettability properties through the creation of micro-niches that protect cells from shear and thus hinder detachment [10,15,37,50–52]. *Escherichia coli* cells are small (typically 1–2 μm in length) and can readily colonize sheltered microenvironments, making their attachment less sensitive to variations in micropattern dimensions and more dependent on the availability of protected microsites for adhesion and biofilm initiation [53,54]. It is reasonable to assume that the smaller feature spacing on D3 created areas of lower local shear that provided a region for bacteria to reside, as evidenced in recent studies on bacterial attachment to surfaces with varying geometries [52].

#### 3.4.2 Antialgal activity.

**Fig 6:**
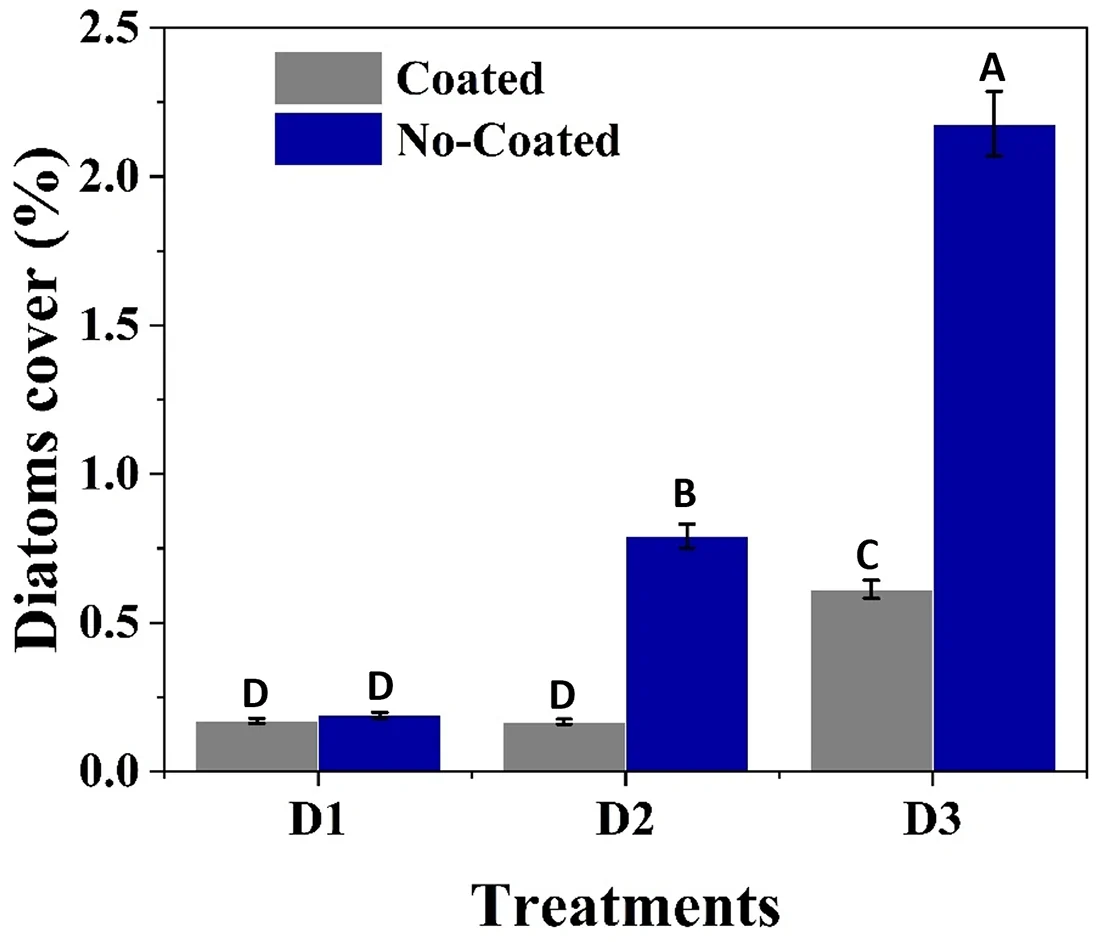
The mean ± SD percentage of cover of the diatom (*Amphora* sp.) on ZnO NRs coated and non-coated D1, D2 and D3 micropatterned samples. Different letters above the bars indicate significant differences by the Tukey HSD test (*P* < 0.05).

Significant variation in the percentage cover of *Amphora* sp. diatoms was observed among treatments and coating conditions (Fig 6). ANOVA results indicated that both ZnO NR coating and treatment (geometry) significantly influenced diatom coverage, and their interaction was also significant (*p* < 0.05) (Table S2 in S2 File). Usually, samples coated with ZnO had higher anti-diatom activity compared to non-coated ones (Fig 6). The highest diatom cover was observed on non-coated D3 samples, whereas the lowest cover was detected on ZnO-coated D1 ZnO and D2 ZnO samples and non-coated D2 samples (Fig 6). An intermediate diatom cover was found on uncoated D2 samples and coated D3 ZnO samples. Tukey’s post hoc analysis revealed significant differences between different treatments (Fig 6).

The lowest diatom coverage was observed on the D1 ZnO and D1 surfaces. Previous studies have demonstrated that diatom settlement is more strongly influenced by surface wettability and hydrodynamic conditions. More hydrophobic surfaces and directional flow can disrupt the formation of adhesive extracellular polymeric substances (EPS), which are essential for diatom adhesion [55,56]. In addition, the diamond geometry of the D2 micropattern alters local flow patterns and increases shear forces at the surface, which would affect diatom adhesion under realistic immersion conditions [57].

Our findings demonstrate that bacteria and diatoms respond differently to micropatterned surfaces. This difference is likely related to their size and attachment mechanisms. Because diatoms are larger than bacteria, their attachment is more strongly influenced by changes in flow direction, shear stress, and surface energy. In contrast, bacterial attachment appears to be affected primarily by the ZnO coating, with the underlying micropattern modulating its effectiveness. Overall, our results demonstrate that the antifouling performance of micropatterned surfaces is species dependent, consistent with previous studies [52,58,59]. The contrasting responses of bacteria and diatoms highlight the importance of multispecies testing when designing effective antifouling surfaces.

### 3.5 Toxicity testing

**Fig 7:**
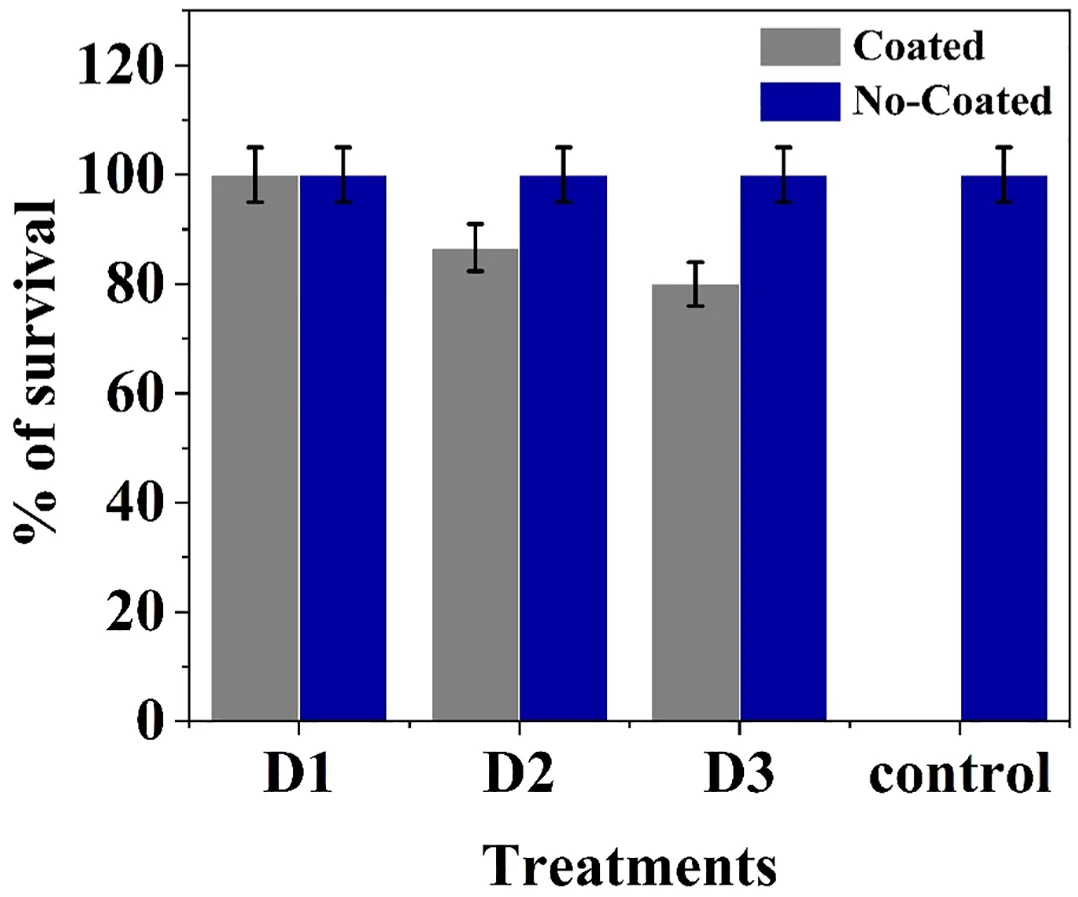
Survival (%) of *Litopenaeus vannamei* larvae after 24 h of exposure to ZnO nanorod-coated and non-coated D1, D2, and D3 micropatterned surfaces. The control consisted of larvae maintained under identical experimental conditions in the absence of any test surface.

Significant variation in shrimp larval survival was observed among treatments after 24 h of exposure (Fig 7). The average percent of alive larvae fluctuated across ZnO NRs-coated treatments but remained relatively stable in all non-coated surfaces. The lowest survival rate (indicating the highest toxicity) occurred in the presence of coated with ZnO D2 and D3 surfaces, whereas the highest survival rates were recorded in all non-coated micropatterned substrates, and the D1 ZnO surfaces (Fig 7).

ANOVA results showed that ZnO NR coatings significantly affected shrimp larval survival (*P* = 0.023), whereas there were no differences between treatments and their interactions (*P* > 0.05) (Table S4 in S2 File). Tukey’s HSD post hoc analysis revealed significant differences between different treatments (Fig 7 and Table S5 in S2 File).

All coated ZnO nanorod surfaces released Zn² ⁺ ions compared to the non-coated control. The highest amount of Zn² ⁺ ions (1.402 ppm) was leached out of the D3 ZnO micropattern surface, which has the highest effective surface area (see section 3.6). However, normalization of Zn² ⁺ release to effective surface area revealed that D3-ZnO exhibited the lowest Zn² ⁺ release per unit surface area compared with D1-ZnO and D2-ZnO (see section 3.6). This indicates that Zn² ⁺ release is not directly proportional to surface area and may also be influenced by nanorod density, morphology, and diffusion pathways.

The release of Zn² ⁺ ions resulted in the toxicity of D2 ZnO and D3 ZnO treatments to shrimp larvae (Fig 7). The dissolved ionic form of zinc (Zn²⁺) plays a critical role in the metabolic functioning of aquatic organisms. However, zinc becomes toxic once elevated levels of this metal are present [60]. Exposure to high levels of Zn² ⁺ has been associated with developmental anomalies, oxidative stress, and detrimental behaviors in the early life stages of aquatic species (e.g., fish and invertebrate larvae) [61–64]. Thus, the potential for ecological harm by the release of Zn²⁺ from ZnO nanoparticles into aquatic systems represents a significant risk factor to the ability of larvae to grow normally and survive in the environment [60,62]. Previous studies suggested that ZnO NRs are less toxic to aquatic organisms than ZnO nanoparticles and ions [19], which demonstrated advantages of using ZnO NRs in antifouling applications. The toxicity assay revealed a significant effect of coating on shrimp larval survival, whereas micropattern geometry and the coating × treatment interaction were not significant. Therefore, the observed toxicity should be primarily attributed to the presence of ZnO NRs coating and its associated physicochemical effects rather than to micropattern geometry itself.

Although the ZnO-coated micropatterned surfaces exhibited relatively low acute toxicity toward *L. vannamei* larvae under the experimental conditions used in this study, these findings should be interpreted with caution. The toxicity assessment was limited to a 24 h acute mortality assay using a single species and did not evaluate sublethal endpoints. Therefore, the present results should be interpreted with caution and further studies involving multiple species, chronic exposure experiments, and sublethal toxicity endpoints are required to fully evaluate the environmental implications of the ZnO-coated micropatterned surfaces.

### 3.6 Leaching test and ROS production

**Fig 8:**
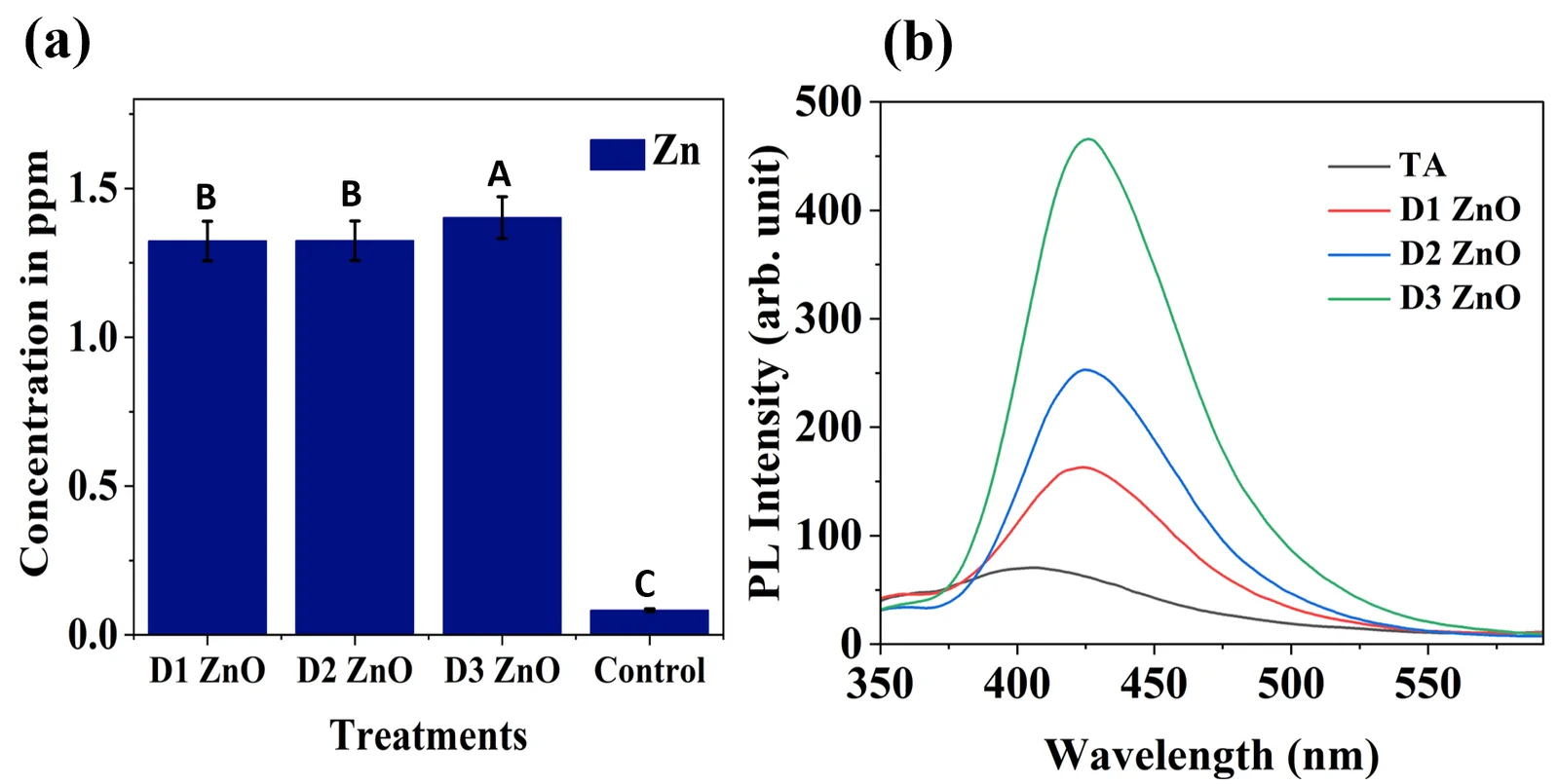
(a) Concentrations (ppm) of Zn^2+^ ions in the presence of ZnO NRs coated (D1 ZnO, D2 ZnO, and D3 ZnO) and non-coated (control, micropattern substrate) micropatterned panels after 24 h. Different letters above the bars indicate significant differences (*P* < 0.05). (b) Photoluminescence emission spectrum of terephthalic acid (TA) reacts with (•OH) produced from the ZnO NRs of D1, D2 and D3 treatments under visible light irradiation. No ROS were produced by the non-coated surfaces (data are not shown).

As expected, Zn^2+^ ions were released from ZnO NRs coatings (Fig 8a). The different treatments had a significant effect on Zn² ⁺ concentration (ANOVA, *P* < 0.05, Table S3 in S2 File). The highest Zn² ⁺ ion concentration was observed for the D3 ZnO sample (1.402 ppm). In comparison, the D1 ZnO and D2 ZnO samples exhibited nearly identical Zn² ⁺ concentrations of 1.324 ppm and 1.325 ppm, respectively. As expected, the non-coated control samples (all micropatterned substrates) showed the lowest Zn² ⁺ concentration (0.08 ppm), confirming minimal background zinc release.

After normalization to the effective surface area, the Zn² ⁺ ion release per unit area was 0.213 ppm/cm² for D1 ZnO, 0.206 ppm/cm² for D2 ZnO, and 0.147 ppm/cm² for D3 ZnO. Tukey’s post hoc test revealed significant differences (*P* < 0.05) between all ZnO-coated and non-coated treatments (Fig 8a). These results confirm that Zn² ⁺ ions were released from the ZnO-coated surfaces. Our previous work using ZnO nanorods deposited on a flat glass substrate showed similar or higher Zn² ⁺ release per surface area than in the current experiment [19–21]. However, direct comparisons between the two studies should be interpreted with caution because the experiments were conducted under different conditions.

The antifouling performance of the tested surfaces was not directly correlated with either Zn² ⁺ release or the acute toxicity of the coatings. For example, D3 ZnO exhibited the highest toxicity but demonstrated the poorest antifouling properties. On the contrary, D1 ZnO showed the highest larval survival rate while exhibiting the highest antifouling activity against both bacteria and diatoms, making this ZnO-coated micropattern a promising antifouling solution. These findings suggest that the antifouling performance is governed by multiple mechanisms rather than by a simple biocidal effect. Although the antibacterial activity is not solely dependent on the release of Zn² ⁺ ions, it is also influenced by the hierarchical micro/nanotopography, the generation of reactive oxygen species (ROS) under light irradiation, and the release of Zn² ⁺ ions into the surrounding medium.

The generation of ROS was confirmed by detecting hydroxyl radicals (•OH) produced from the three ZnO nanorod (NR)-coated micropatterned surfaces using photoluminescence (PL) measurements (Fig 8b). The PL intensity is directly proportional to the amount of •OH generated. In contrast, no or only negligible ROS production was detected on the non-coated (control) surfaces. The PL emission results showed that the D3 ZnO surface generated a higher amount of •OH than the D1 ZnO and D2 ZnO surfaces (Fig 8b). Overall, the generation of •OH supports the activity of the ZnO nanorod-coated micropatterned surfaces against bacteria, diatoms, and shrimp larvae. Scavenger (radical trapping) experiments to confirm ROS generation by ZnO nanorods have been extensively reported in the literature [65,66]. Therefore, they were not repeated in the present study.

It is well known that semiconductor materials, such as ZnO NR-coated surfaces, release reactive oxygen species (•OH, O_2_•^−^, and H_2_O_2_) under light irradiation. The generation of these ROS is considered one of the principal mechanisms responsible for the antifouling activity of ZnO-coated surfaces [20]. ROS possess well-documented antimicrobial properties [67].

Our results suggest that the moderate ROS production and Zn² ⁺ ion release observed for the D1 ZnO and D2 ZnO surfaces were sufficient to inhibit bacterial and diatom attachment and growth. Specifically, photoexcitation of ZnO nanostructures generates ROS that induce oxidative damage to microbial cell membranes. This membrane damage increases membrane permeability, leading to the leakage of intracellular components and reducing the ability of bacteria and diatoms to attach to and colonize the surface [29,68]. In addition to membrane damage, ROS can cause oxidative degradation of important biomolecules, such as lipids, proteins, and DNA, thereby disrupting normal cellular functions and ultimately causing cell death [69].

Therefore, the primary antifouling mechanism provided by ZnO coatings is the generation of ROS, which creates an environment detrimental to biofilm formation through the induction of oxidative stress in attached organisms [2,69]. As a result, the increased production of ROS from the ZnO-coated micropatterned surfaces likely contributed to the reduced bacterial and diatom colonization observed in this study. Overall, the antifouling performance of the tested surfaces is likely the result of several complementary mechanisms, including ROS generation, controlled Zn² ⁺ release, surface wettability, and surface microtopography.

### 3.7 Post characterization

**Fig 9:**
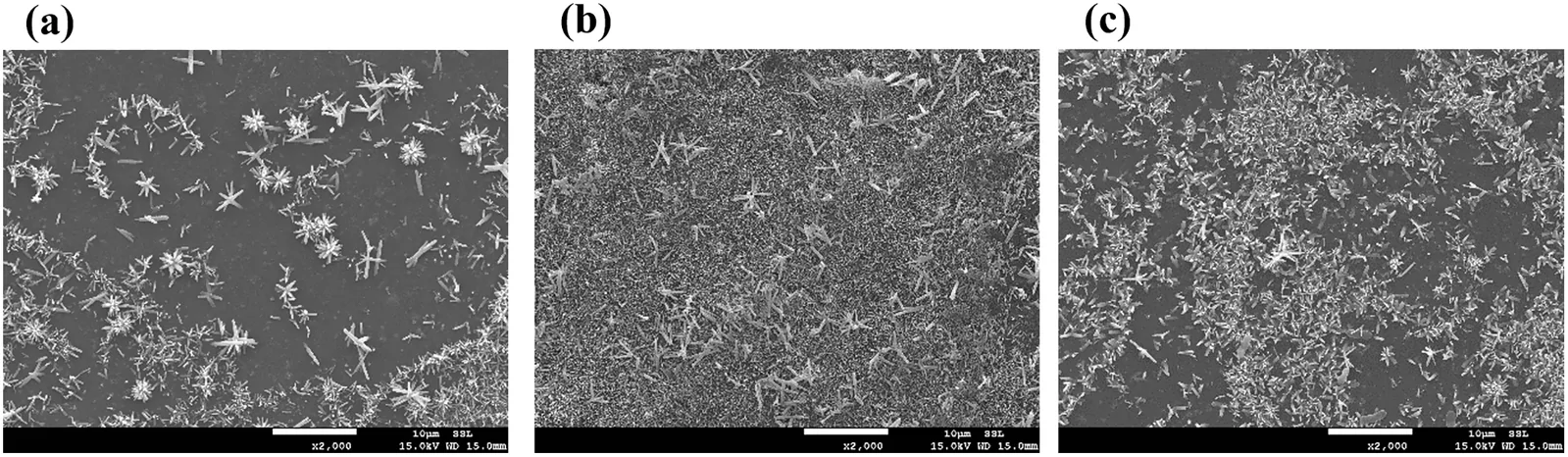
SEM images of post characterization of (a) D1 ZnO, (b) D2 ZnO, and (c) D3 ZnO.

Post-characterization SEM images of the D1 ZnO, D2 ZnO, and D3 ZnO surfaces after the antibacterial assays are shown in Fig 9. The ZnO nanorods remained clearly visible on all tested surfaces, indicating that they retained their structural integrity throughout the biological experiments. This stability is likely attributable to the minimal photodissolution of ZnO nanorods under visible light irradiation. In addition, the antibacterial assays were conducted under neutral pH conditions, where ZnO is relatively stable, and in a nutrient medium with high concentrations of Ca² ⁺ , K⁺ and Na⁺ ions, which may further reduce ZnO dissolution.

A small amount of bacterial attachment was observed on the ZnO nanorod-coated surfaces (Fig S1 in S1 File). This may be associated with the light-induced transition of the ZnO-coated micropatterned surfaces from a superhydrophobic to a more hydrophilic state, which could facilitate limited bacterial adhesion. However, this hypothesis requires further investigation, and additional experiments are needed to confirm the underlying mechanism.

Overall, the post-characterization SEM observations demonstrate that the ZnO nanorod coating remained structurally stable after the antifouling assays. The preservation of the nanorod morphology, together with the relatively low Zn² ⁺ release, indicates that the coating maintained its integrity under the experimental conditions. These findings suggest that the ZnO-coated micropatterned surfaces possess good structural stability while retaining their antifouling activity, making them promising candidates for prolonged antifouling applications. Nevertheless, further studies under long-term marine exposure are required to evaluate the durability and performance of the coatings under realistic environmental conditions.

## 4. Conclusions

Among all tested micropatterned polymers, the D1 ZnO and D2 ZnO surfaces exhibited the most effective antifouling performance, with the lowest levels of bacterial and diatom adhesion. These results indicate that the D1 ZnO and D2 ZnO geometries provide an optimal combination of surface structure and physicochemical properties, particularly their high water contact angles. However, considering the acute toxicity results, D1 ZnO appears to be the most suitable candidate for future antifouling applications, as it combined excellent antifouling performance with the highest larval survival.

The enhanced antifouling performance of the D1 ZnO and D2 ZnO surfaces is likely attributable to the combined effects of reactive oxygen species (ROS) generation, Zn² ⁺ ion release, surface wettability, and the hierarchical micro/nanotopography. The results suggest that the combination of ZnO coating and micropatterned topography improves antifouling performance. However, because a flat ZnO-coated control was not included, the individual contributions of the ZnO coating and surface topography could not be fully distinguished. Therefore, the proposed synergistic effect should be regarded as preliminary and warrants further investigation.

In contrast, the D3 and D3 ZnO surfaces exhibited the weakest antifouling performance, characterized by increased bacterial and diatom colonization and lower larval survival, which may be associated with their lower water contact angles and different surface geometries. Post-characterization SEM observations demonstrated that the ZnO nanorod coatings remained structurally intact after the biological assays, indicating good coating stability under the experimental conditions.

Overall, ZnO-coated micropatterned surfaces represent a promising approach for marine antifouling applications. Nevertheless, further research is needed to evaluate their long-term durability under natural conditions, quantify the individual contributions of ZnO coatings and surface topography, and assess chronic and sublethal toxicity across multiple marine species before practical applications can be recommended.

## Supporting information

S1 File Figure S1. Magnified image of bacteria on ZnO nanorods coated micropatterned D1 (a) and D3 (b) surfaces. Bacteria were not detected on ZnO nanorods coated micropatterned D2 surface.
(DOCX)

S2 File Tables S1–S5: Supplementary figures showing ANOVA test results among the treatments. (DOCX)

S3 File Bacterial, Diatoms and Zn data. (XLSX)
