# Supplementary material for: Antifouling properties of ZnO nanorods coating on micropatterned polymers
Source: PLoS One. 2026 Sep 18;21(9):e0357826. doi: 10.1371/journal.pone.0357826 (PMC13588403; doi:10.1371/journal.pone.0357826)
Supplement: Supplementary file 1 [file pone.0357826.s001.docx]

**Supplementary Figures**

**Figure S1.** Magnified image of bacteria on ZnO nanorods coated micropatterned D1 (a) and D3 (b) surfaces. Bacteria were not detected on ZnO nanorods coated micropatterned D2 surface.

**(a)**

**(b)**
