# Supplementary material for: Antifouling properties of ZnO nanorods coating on micropatterned polymers
Source: PLoS One. 2026 Sep 18;21(9):e0357826. doi: 10.1371/journal.pone.0357826 (PMC13588403; doi:10.1371/journal.pone.0357826)
Supplement: Supplementary file 2 [file pone.0357826.s002.docx]

**Supplementary Tables**

**Table S1:** ANOVA results comparing the bacterial density (cells mm^-2^) on ZnO NRs coated and non-coated micropatterned polymers patterns (D1, D2 and D3). Factors are coating (with ZnO and without it) and treatments (micropatterns D1-D3). Significant values (*p*<0.05) are in bold.

| Source of variation | Df | Sum of Squares | F Ratio | P-value |
| --- | --- | --- | --- | --- |
| **Coating** | **1** | **1.07E+08** | **20.2304** | **<0.0001** |
| Treatment | 2 | 29295444 | 2.7766 | 0.065 |
| **Coating x Treatment** | **2** | **55216333** | **5.2335** | **0.0062** |

**Table S2.** ANOVA results comparing the diatoms cover (%) on ZnO NRs coated and non-coated micropatterned polymers patterns (D1, D2 and D3) panels. Factors are coating (with ZnO and without it) and treatments (micropatterns D1-D3). Significant values (*p*<0.05) are in bold.

| *Source of variation* | *Df* | *Sum of Squares* | *F Ratio* | *P-value* |
| --- | --- | --- | --- | --- |
| **Coating** | **1** | **2.518868** | **12.7915** | **0.0038** |
| **Treatments** | **2** | **4.638301** | **11.7773** | **0.0015** |
| **Coating x Treatments** | **2** | **1.923027** | **4.8828** | **0.0281** |

**Table S3.** ANOVA test comparing the shrimp survival in the presence of ZnO NRs coated and non-coated D1, D2, and D3 micropatterned samples. Factors are coating (with ZnO and without it) and treatments (micropatterns D1-D3). Significant values (*p*<0.05) are in bold.

| Source | Df | Sum of Squares | F Ratio | P- value |
| --- | --- | --- | --- | --- |
| **Coating** | **1** | **555.5556** | **6.25** | **0.0279** |
| Treatments | 2 | 311.1111 | 1.75 | 0.2153 |
| Coating x Treatments | 2 | 311.1111 | 1.75 | 0.2153 |

**Table S4.** ANOVA test comparing the concentrations (ppm) of Zn^2+^ ions in the presence of ZnO NRs coated (D1 ZnO, D2 ZnO, and D3 ZnO) and non-coated (control) micropatterned panels (samples) after 24 h. Significant values (*p*<0.05) are in bold.

| *Source* | *Df* | *Sum of Squares* | *F Ratio* | *P-value* |
| --- | --- | --- | --- | --- |
| **Samples** | **3** | **3.6250667** | **4.662e+8** | **<0.0001** |

**Table S5.** Tukey's HSD post-hoc comparisons of Zn²⁺ ion release among treatments.

| *Comparison* | *p-value* | *Significant* |
| --- | --- | --- |
| **Control vs D1** | **< 0.001** | **Yes** |
| **Control vs D2** | **< 0.001** | **Yes** |
| **Control vs D3** | **< 0.001** | **Yes** |
| D1 vs D2 | 0.994 | No |
| **D1 vs D3** | **0.002** | **Yes** |
| **D2 vs D3** | **0.002** | **Yes** |
